# Acute Rhabdomyolysis as a Rare Manifestation of Hypothyroidism: A Case Report and Literature Review

**DOI:** 10.7759/cureus.86675

**Published:** 2025-06-24

**Authors:** Nada Benabdelmalek, Ali Halouache, Lhoussaine Abainou, Saadia Ait Malek, Mariam Erraoui, Imad Ghozlani

**Affiliations:** 1 Department of Rheumatology, Hassan 2 Hospital, University Hospital of Souss Massa, Agadir, MAR; 2 Department of Endocrinology, Oued Eddahab Military Hospital, Agadir, MAR; 3 Cartilage and Bone (CARBONE) Research Team, Research Laboratory of Innovation in Health Sciences (LARISS), Faculty of Medicine and Pharmacy of Agadir, Ibn Zohr University, Agadir, MAR; 4 Department of Rheumatology, Oued Eddahab Military Hospital, Agadir, MAR

**Keywords:** case report, hypothyroidism, myopathy, rare manifestation, rhabdomyolysis, thyroid dysfunction

## Abstract

Thyroid myopathy is often subtle, but massive rhabdomyolysis secondary to hypothyroidism is an exceptional phenomenon, with approximately 30 to 40 cases reported in the medical literature to date. We report a case of rhabdomyolysis secondary to profound hypothyroidism in the absence of any precipitating factor. This is a 56-year-old woman who underwent a right mastectomy six years ago for breast cancer and received adjuvant chemotherapy for two months followed by four weeks of radiotherapy. She has been on letrozole 2.5 mg daily for six years. Over the past three months, she has developed physical and psychological asthenia, constipation, muscle fatigue, and myalgia, predominantly in the proximal muscles of the four limbs. Clinical examination found signs of myxedema infiltration and signs of hypometabolism.

The laboratory assessment revealed elevated levels of total creatine phosphokinase (CPK), lactate dehydrogenase (LDH), and transaminases (aspartate aminotransferase (AST) and alanine aminotransferase (ALT)). The thyroid function test indicated profound hypothyroidism, with a significantly elevated ultrasensitive thyroid-stimulating hormone (TSHus) level. Anti-thyroperoxidase antibodies were negative, as were anti-thyroglobulin antibodies. Cervical ultrasound revealed an atrophic thyroid and loss of the musculoparenchymal gradient, suggestive of thyroiditis. Computed tomography brain was normal. The diagnosis was made based on her clinical presentation and laboratory tests. The outcome was marked, after treatment, by good clinical and biological improvement.

## Introduction

Hypothyroidism is one of the most common endocrine disorders in adults, resulting from a deficiency of thyroid hormones essential for the regulation of cellular metabolism. It can be primary (affecting the thyroid gland itself) or secondary (of pituitary or hypothalamic origin), the primary form being the most widespread, particularly in the context of Hashimoto's autoimmune thyroiditis. Epidemiology indicates a prevalence of up to 5% in certain adult populations, with a marked female predominance [1,2]. 

The clinical presentation of hypothyroidism is often insidious. It is the cause of a set of clinical and biological anomalies including hypometabolism syndrome: asthenia, chilliness, bradycardia, constipation, dry skin, psychomotor slowing, cognitive disorders, and even depression. In a more advanced stage, it can lead to myxedema, heart failure, neurological disorders, and hematological disorders [3].

Thyroid myopathy is often subtle and usually consists of muscle weakness, some cramps, and sometimes myalgia. Among the rare but severe complications, rhabdomyolysis deserves special attention. This entity is defined by an acute lysis of skeletal muscle fibers resulting in the release into the bloodstream of muscle enzymes such as creatine phosphokinase (CPK), myoglobin and lactate dehydrogenase (LDH). The biological picture can range from a simple elevation of CPK to major complications such as acute renal failure secondary to myoglobinuria [4]. Rhabdomyolysis is most often reported in traumatic, toxic or metabolic contexts (excessive muscle strain, statin use, sepsis, hypokalemia, seizures). However, its association with hypothyroidism is uncommon, often unrecognized, but well documented in the literature in the form of clinical cases [5,6].

We report a case of moderate rhabdomyolysis secondary to profound hypothyroidism, aiming to emphasize this rare but clinically important association.

## Case presentation

This is a 56-year-old female patient who had a right mastectomy six years ago for breast cancer and received adjuvant chemotherapy for two months, followed by radiotherapy completed in four weeks. She had been receiving letrozole 2.5 mg daily for six years. There was no known personal or family history of autoimmune disease. Over the past three months, she reported physical and psychological asthenia, along with constipation, muscle fatigue, and myalgia, predominantly affecting the proximal muscles of all four limbs.

The clinical examination revealed an asthenic patient who was normotensive, with a blood pressure of 106/65 mmHg, and a heart rate of 73 beats per minute, consistent with normal sinus rhythm. Her weight had increased to 94 kg from a baseline of 82 kg over the preceding three-month period, corresponding to the duration of her symptoms. She exhibited a depressed affect and marked psychomotor retardation.

She presented with dry, cold skin, a pale complexion, sparse eyebrows, axillary and pubic alopecia and brittle nails. Muscle strength was decreased in all four limbs, particularly at the proximal regions. There was pain on palpation and mobilization of the left shoulder, along with dorsal paravertebral contracture and reduced osteotendinous reflexes. Cervical examination found skin of normal color, the thyroid was not palpable, without tenderness or swelling on palpation, locoregional examination did not find any lymphadenopathy.

The biological assessment found total CPK at 1084 IU/l, LDH at 399 U/L, aspartate aminotransferase (AST) at 95 IU/l and alanine aminotransferase (ALT) at 65 IU/l. The thyroid assessment showed profound hypothyroidism: the thyroid-stimulating hormone (TSH) level was at 71.46 mIU/l. The emergency electrocardiogram showed a regular sinus rhythm without microvoltage.

The rest of the assessment revealed creatinine level at 8.29 mg/l and urea at 0.36 g/l, normochromic normocytic anemia with a hemoglobin level of 11.2 g/dL, with normal ferritin level at 74 ng/ml, lymphopenia at 884/mm3 and slight neutropenia at 1938/mm3.

The lipid profile revealed dyslipidemia with a total cholesterol level of 4.05 g/l, an increased low-density lipoprotein cholesterol (LDLc) of 3.01 g/l, an increased high-density lipoprotein cholesterol (HDLc) of 0.70 g/l and increased triglycerides of 1.70 g/l.

Natremia, kalemia, chloremia, bicarbonates, fasting blood glucose and 8:00 a.m. serum cortisol level were normal. Anti-thyroperoxidase antibodies were negative at 0.03 IU/ml, as were anti-thyroglobulin antibodies (Table 1).

**Table 1:** Laboratory Test Results with Reference Ranges CPK: creatine phosphokinase, LDH: lactate dehydrogenase, AST: aspartate aminotransferase, ALT: alanine aminotransferase, TSH: thyroid stimulating hormone, LDLc: low-density lipoprotein cholesterol, HDLc: high-density lipoprotein cholesterol

	Laboratory test results	Reference ranges
CPK	1084 IU/l	24 – 195 IU/l
LDH	399 IU/l	125 – 220 IU/l
AST	95 IU/l	0 – 35 IU/l
ALT	65 IU/l	0 – 40 IU/l
TSH (Ultrasensitive)	71.460 mIU/l	0.4 – 4.94 mIU/l
Urea	0.36 g/l	0.35 – 0.45 g/l
Creatinine	8.29 mg/l	5 – 12 mg/l
Hemoglobin	11.2 g/dL	12.0 – 15.5 g/dL
Ferritin	74 ng/ml	15 – 150 ng/mL
Lymphocyte	884/mm3	1,000 – 4,000/mm³
Neutrophilic granulocytes	1938/mm3	2,000 – 7,500/mm³
Total cholesterol	4.05 g/l	2 – 2.39 g/L
LDLc	3.01 g/l	< 1.0 g/l
HDLc	0.70 g/l	> 0.40 g/l
Triglycerides	1.70 g/l.	< 1.50 g/l
Natremia	139 mEq/l	135 – 145 mEq/l
Kalemia	4.4 mEq/l	3.6 – 5.1 mEq/l
Chloremia	102 mmol/l	95 – 105 mmol/l
Bicarbonates	26 mEq/l	23 – 27 mEq/l
Fasting blood glucose	0,76 g/l	0,70 – 1,05 g/l
8 a.m. serum cortisol level	193.10 nmol/l	101.2 – 535.7 nmol/l
Anti-thyroperoxidase antibodies	0.03 IU/ml	< 5.6 IU/ml
Anti-thyroglobulin antibodies	0.05 IU/ml	< 4.0 IU/mL

**Figure 1:**
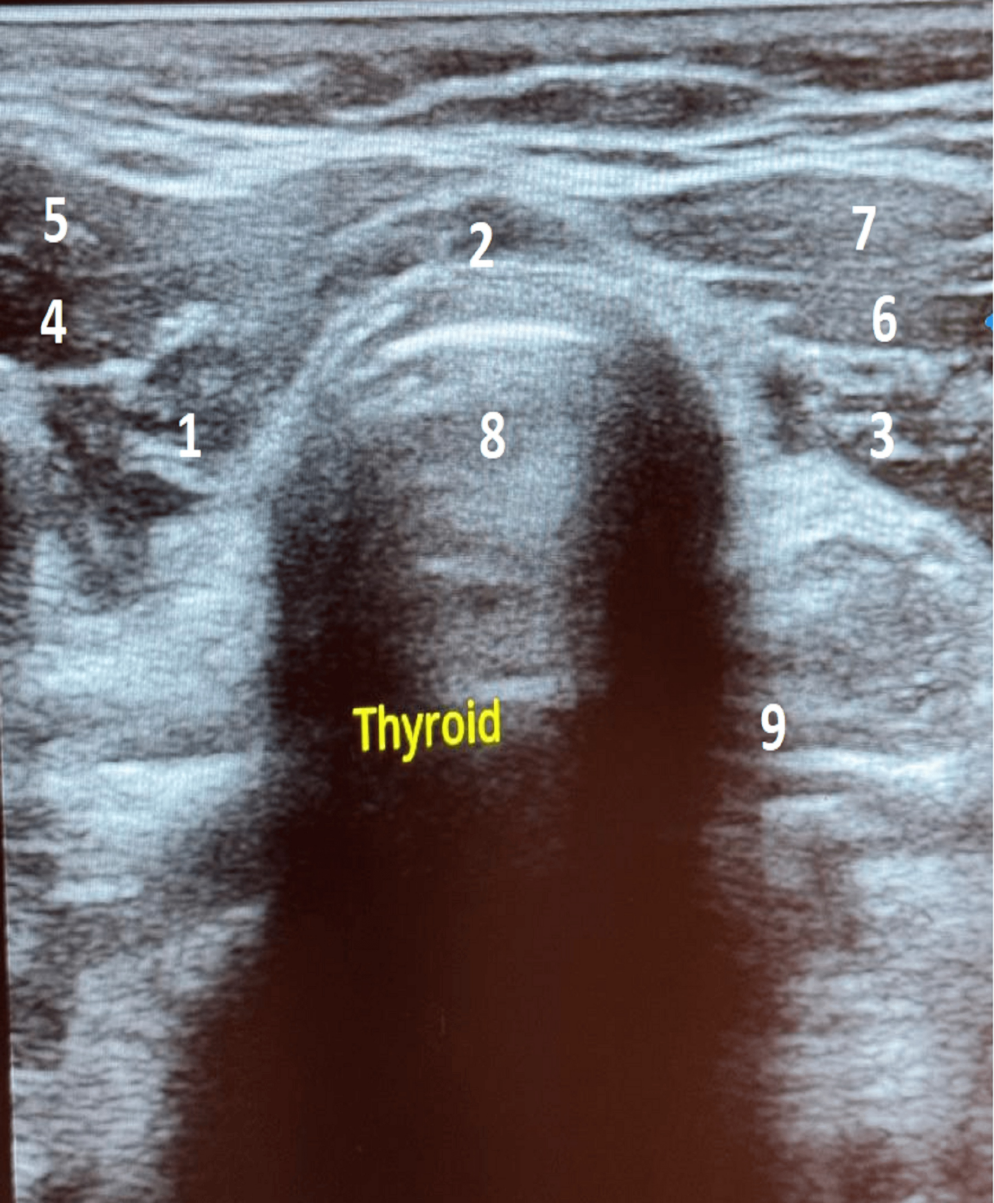
Cervical ultrasound in transverse section Cervical ultrasound in transverse section showing a reduced thyroid size, no identifiable nodules, with loss of the musculoparenchymal gradient suggesting thyroiditis: 1. Right lobe of the thyroid gland, 2. Isthmus, 3. Left lobe of the thyroid gland, 4. Right omohyoid muscle, 5. Right sternothyroid muscle, 6. Left sternohyoid muscle, 7. Left sternothyroid muscle, 8. Trachea, 9. Esophagus

**Figure 2:**
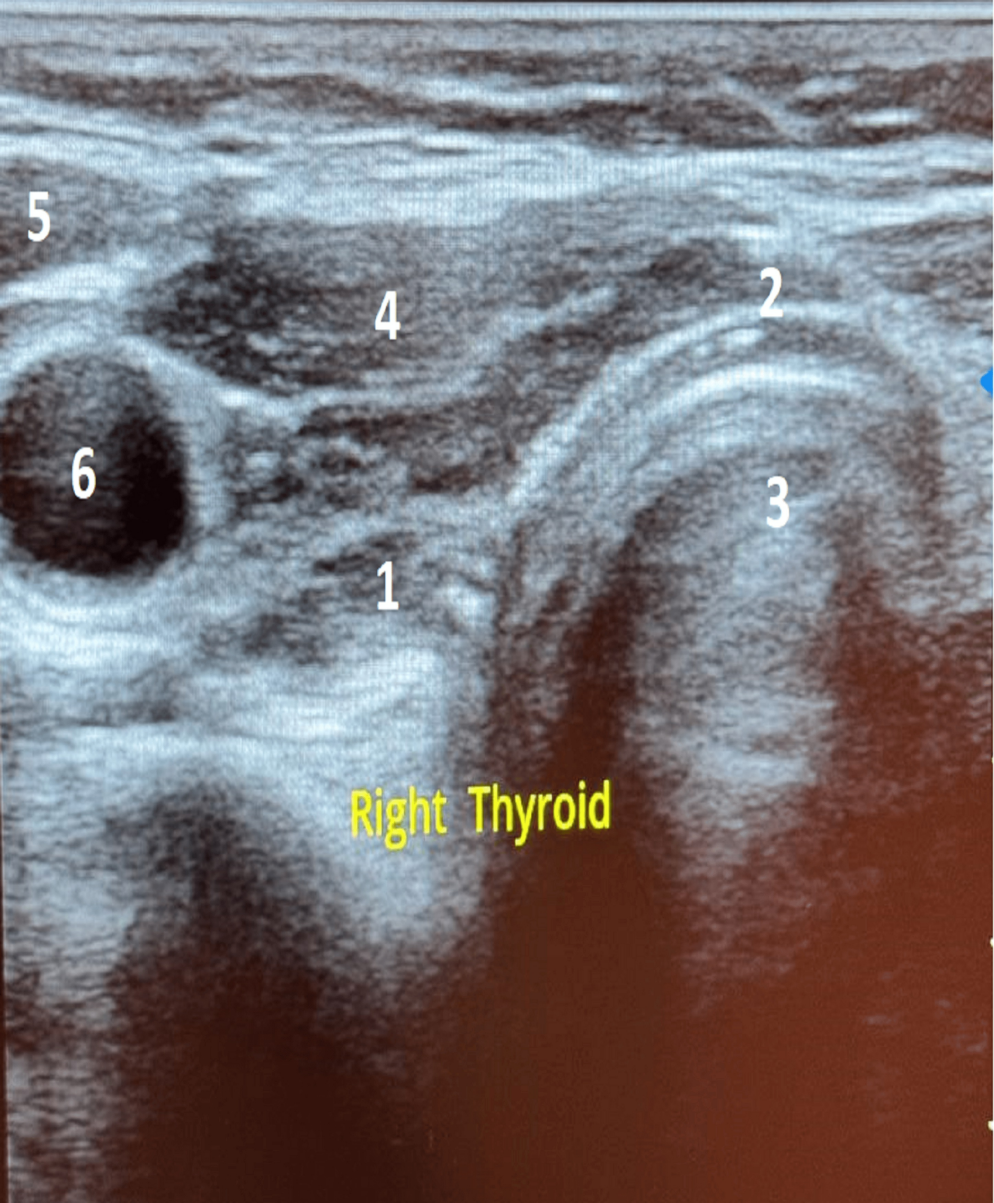
Transverse section of the right lobe of the thyroid gland 1. Right lobe, 2. Isthmus, 3. Trachea, 4. Right sternothyroid and sternohyoid muscles, 5. Right sternocleidomastoid muscle, 6. Right common carotid artery

**Figure 3:**
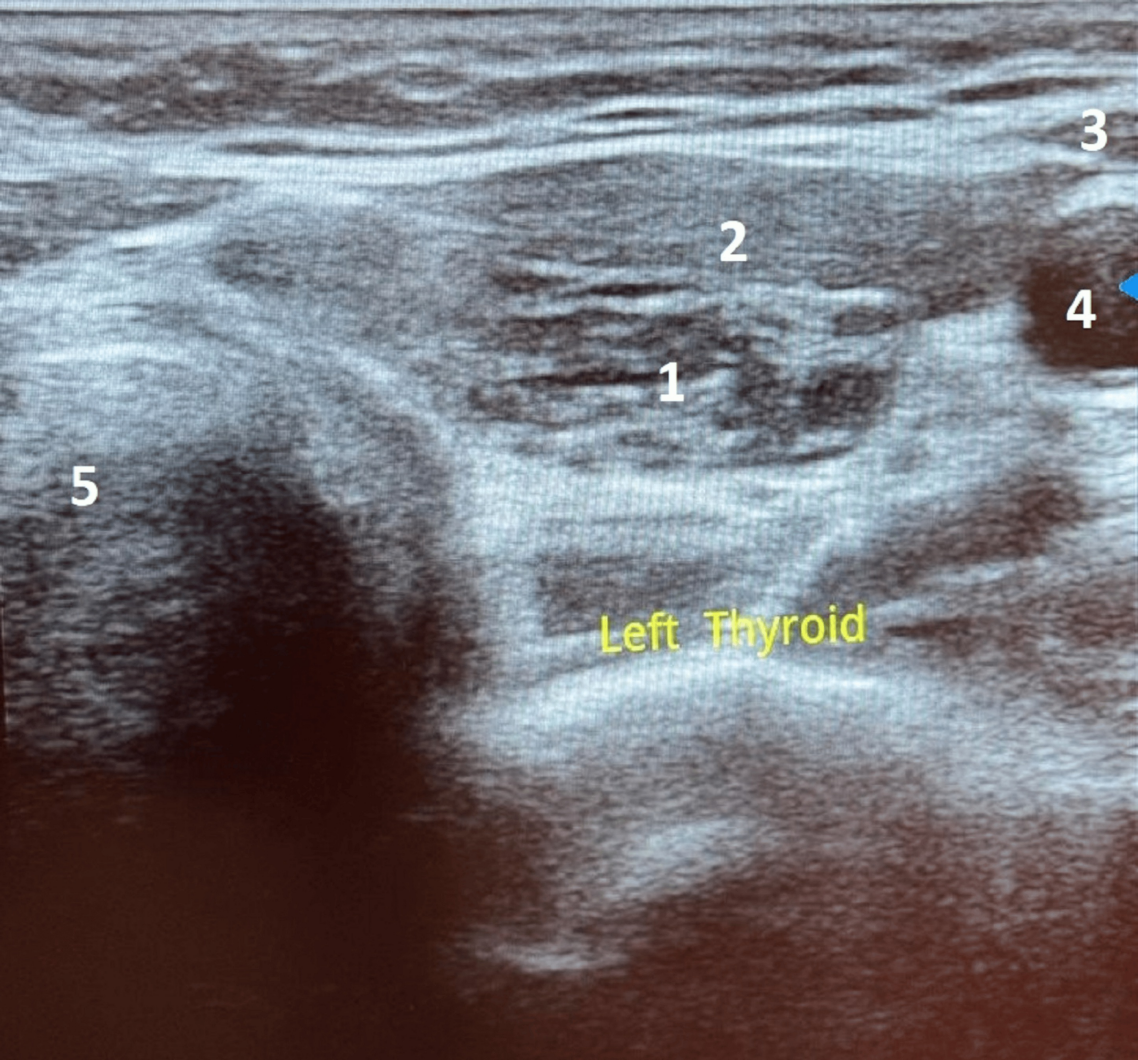
Transverse section of the left lobe of the thyroid gland 1. Left lobe, 2. Left sternothyroid and sternohyoid muscles, 3. Left sternocleidomastoid muscle, 4. Left common carotid artery, 5. Trachea

Cervical ultrasound revealed a reduced thyroid size without any identifiable nodule with loss of the musculoparenchymal gradient, suggesting thyroiditis (Figures 1, 2, 3).

In the presence of altered consciousness, a brain CT scan was performed to rule out myxedematous cerebral edema and was found to be normal. Musculoskeletal ultrasound of the left shoulder, conducted due to scapular pain, revealed signs of chronic tendinopathy of the subscapularis and supraspinatus, along with acromioclavicular synovitis, with no Doppler abnormalities. Chest X-ray was normal.

Therapeutically, the patient was put on slow and progressive replacement therapy - under daily ECG monitoring - based on levothyroxine, starting with 25 ug/day combined with corticosteroid therapy based on hydrocortisone 10 mg/day as a preventive measure to avoid acute adrenal insufficiency. This approach is consistent with current guidelines, which recommend initiating empiric glucocorticoid therapy prior to thyroid hormone replacement in cases of severe hypothyroidism, in order to prevent a potential adrenal crisis, even in the absence of confirmed adrenal insufficiency.

The evolution was marked by a good clinical and biological improvement with weight loss, disappearance of myxedema and normalization of TSH to 0.5 mIU/l, CPK to 61 IU/l, LDH to 190 IU/l, AST to 20 IU/l, and ALT to 9 IU/l.

## Discussion

Muscle involvement is a frequent clinical manifestation of symptomatic hypothyroidism, manifesting in particular by myalgia, stiffness, muscle cramps and marked asthenia [7]. However, rhabdomyolysis, defined as a process of acute lysis of striated muscle fibers with massive release of intracellular constituents such as CPK, myoglobin, potassium, and various muscle enzymes into the systemic circulation, remains a rare but potentially fatal complication of hypothyroidism [8-10]. Clinically, rhabdomyolysis typically presents with myalgia, muscle cramps, post-exertional stiffness, and in some cases, pseudomyotonic phenomena. This condition has a clear male predominance, with an incidence four times higher in men than in women, and occurs primarily between the ages of 20 and 60 [11,12]. However, our case highlights that rhabdomyolysis secondary to hypothyroidism can also occur in female patients, despite this epidemiological trend. Although less frequent, such cases are well documented in the literature. This reinforces the need for clinicians to maintain a high index of suspicion, even in patients who do not fit the usual demographic profile, and to consider hypothyroidism in the differential diagnosis of unexplained rhabdomyolysis regardless of sex.

Genetic data suggest an increased susceptibility to rhabdomyolysis in individuals of Japanese descent, due to specific mutations in genes involved in muscle metabolism, such as HADHA, HADHB, CACNA1S, and HLA-DRB1* 04:06 [13]. An elevation of serum CPK concentration to at least five times the normal value is sufficient to establish the diagnosis of rhabdomyolysis.

Before retaining the diagnosis of hypothyroid myopathy in a context of rhabdomyolysis, it is essential to eliminate the other classic causes reported in the literature [9]. First, there are traumatic causes, including crush syndrome, muscle contusions, burns, and electrocutions.

Prolonged muscle compression - such as that associated with the use of anti-shock trousers or improper intraoperative positioning - as well as vascular obstruction, represent important etiologies of rhabdomyolysis due to ischemia-reperfusion injury [14]. Causes related to excessive muscular exertion include intense physical exercise [15], convulsions and tetanus crisis. Viral infections, such as influenza or HIV, as well as bacterial infections such as Legionnaires' disease, streptococcal disease, tularemia, and salmonellosis, are also common causes of rhabdomyolysis. Fungal and parasitic infections, including malaria, should also be considered during diagnostic evaluation [16]. Drug-related causes include statins, fibrates, neuroleptics, corticosteroids, muscle relaxants, and salicylates, which are commonly associated with an increased risk of rhabdomyolysis. Toxic substances, such as alcohol, heroin, cocaine, amphetamines, ethylene glycol, carbon monoxide, and certain venoms (especially those from snakes), are also contributing factors [14]. Metabolic and electrolyte disorders, including hyperosmolarity, hypokalemia, hypophosphatemia, and dysnatremia, should also be investigated. Electrolyte disorders can cause serious disturbances of muscle function [17]. Finally, certain situations associated with hyperthermia, such as neuroleptic malignant syndrome, malignant hyperthermia [18], peranesthetic reactions and heat stroke, as well as inflammatory muscle diseases [19] such as polymyositis and dermatopolymyositis, may also be involved in the occurrence of rhabdomyolysis. In our observation, all of these etiologies were systematically explored and none were identified, which reinforces the probability of a hypothyroid origin of rhabdomyolysis.

The exact mechanism of rhabdomyolysis in hypothyroidism is not fully understood.

Hypothyroidism-associated myopathy results primarily from impaired muscle energy metabolism, particularly mitochondrial oxidative metabolism, thereby compromising ATP production essential for muscle contraction and function [20]. Furthermore, a dysfunction of glycogen metabolism was observed, characterized by a decrease in the activity of glycolytic enzymes, reducing the ability of the muscle to meet the basic energy demand [7]. These metabolic abnormalities can be explained in particular by the crucial role of thyroid hormones in the transcriptional regulation of numerous muscle genes, influencing the differentiation of myocytes and the expression of contractile proteins [21]. This deregulation leads to structural fragility of muscle fibers, resulting in long-term atrophy of type II fibers, which are more dependent on glycogenolysis, and compensatory hypertrophy of type I fibers. Biochemical studies have also highlighted qualitative and quantitative alterations of myosin, a reduction in the activity of the Na⁺/K⁺-ATPase pump, as well as a significant decrease in intramuscular carnitine concentrations [22,23].

Furthermore, the hematological disorders observed in our patient - ​​normochromic normocytic anemia, mild neutropenia and lymphopenia - are common in cases of severe hypothyroidism. The anemia is multifactorial, attributed to a decrease in erythropoietin production, a slowdown in bone marrow maturation, or even concomitant malabsorption (particularly of iron or folates) [24]. These abnormalities, although moderate, contribute to the overall clinical picture of hypometabolism.

The management is based on hormone replacement therapy with Levothyroxine, administered in progressive doses according to cardiovascular tolerance. Close monitoring of thyroid, muscle (CPK), renal (creatinine) and hematological assessment is essential. The outcome is generally favorable under replacement therapy, with progressive normalization of biological parameters. and the disappearance of signs of hypothyroidism in a few weeks to months [25].

Our observation illustrates the importance of considering hypothyroidism as a potential, albeit rare, cause of rhabdomyolysis, especially in situations where no obvious triggering factor is present. A simple thyroid assessment allows for rapid diagnosis and effective treatment, thus preventing severe complications.

## Conclusions

This clinical case highlights a rare but significant complication of profound hypothyroidism, which is rhabdomyolysis. Although rare, the association between these two entities is clinically significant, particularly due to the risk of severe complications such as acute renal failure, hyperkalemia, cardiac dysfunction, and metabolic acidosis.

Moderate biological rhabdomyolysis, without obvious cause, should alert clinicians to consider thyroid assessment, even in the absence of classic signs of hypothyroidism, which are often not spontaneously reported.

Rapid initiation of hormone therapy based on Levothyroxine constitutes the pivot of treatment, with favorable progress generally observed in both muscular and hematological parameters.
